# Integrated analysis of the ubiquitination mechanism reveals the specific signatures of tissue and cancer

**DOI:** 10.1186/s12864-023-09583-z

**Published:** 2023-09-04

**Authors:** Deyu Long, Ruiqi Zhang, Changjian Du, Jiapei Tong, Yu Ni, Yaqi Zhou, Yongchun Zuo, Mingzhi Liao

**Affiliations:** 1 Center of Bioinformatics, College of Life Sciences, Northwest A&F University, 712100 Yangling, Shaanxi China; 2 State Key Laboratory of Reproductive Regulation and Breeding of Grassland Livestock, College of Life Sciences, Inner Mongolia University, 010070 Hohhot, China; 3 College of Information Engineering, Northwest A&F University, Yangling, Shaanxi China

**Keywords:** Ubiquitination regulator, Integrative analysis, Spermatogenesis, Cancer, Prognostic

## Abstract

**Background:**

Ubiquitination controls almost all cellular processes. The dysregulation of ubiquitination signals is closely associated with the initiation and progression of multiple diseases. However, there is little comprehensive research on the interaction and potential function of ubiquitination regulators (UBRs) in spermatogenesis and cancer.

**Methods:**

We systematically characterized the mRNA and protein expression of UBRs across tissues and further evaluated their roles in testicular development and spermatogenesis. Subsequently, we explored the genetic alterations, expression perturbations, cancer hallmark-related pathways, and clinical relevance of UBRs in pan-cancer.

**Results:**

This work reveals heterogeneity in the expression patterns of UBRs across tissues, and the expression pattern in testis is the most distinct. UBRs are dynamically expressed during testis development, which are critical for normal spermatogenesis. Furthermore, UBRs have widespread genetic alterations and expression perturbations in pan-cancer. The expression of 79 UBRs was identified to be closely correlated with the activity of 32 cancer hallmark-related pathways, and ten hub genes were screened for further clinical relevance analysis by a network-based method. More than 90% of UBRs can affect the survival of cancer patients, and hub genes have an excellent prognostic classification for specific cancer types.

**Conclusions:**

Our study provides a comprehensive analysis of UBRs in spermatogenesis and pan-cancer, which can build a foundation for understanding male infertility and developing cancer drugs in the aspect of ubiquitination.

**Supplementary Information:**

The online version contains supplementary material available at 10.1186/s12864-023-09583-z.

## Background

Protein ubiquitination is involved in the regulation of various biological processes, such as enzyme activity, autophagy, cell cycle progression, cell proliferation, protein degradation, endogenous protein stability, signal transduction, innate and adaptive immune responses, inflammatory response, and DNA damage response [1–5]. It is a dynamic and reversible post-translational modification mediated by UBRs. Based on their functions, UBRs are classified into writers (adding ubiquitin to substrates), readers (recognizing modified proteins) and erasers (removing ubiquitin from substrates) [6, 7]. Among them, the E1 ubiquitin-activating enzyme (E1), E2 ubiquitin-conjugating enzyme (E2) and E3 ubiquitin-ligating enzyme (E3) are writers; numerous proteins with ubiquitin and ubiquitin-like binding domains (UBDs) or ubiquitin-like domains (ULDs) are readers, and deubiquitinases (DUBs) are erasers [2, 8–11]. The abnormal function of UBRs is one of causes of developmental disorders and cancers [12]. Therefore, systematically investigating the functions of UBRs in the development and cancer could provide new strategies for therapeutic intervention.

UBRs are essential in tissue development and dysregulated in various diseases. For example, E3s play a crucial role in intricate cellular signaling networks that guide embryonic development, which include retinoic acid, growth factors, Hedgehog, Wnt/β-catenin, cyclin-dependent kinases and many other vital molecules [13]. Alterations in the activity of many E3s are markedly correlated with the etiology of malignant tumors in humans, and their mutations may contribute to the dysregulation of tumor suppressors or deficiency of ubiquitination of oncogenic proteins [14–16]. Importantly, some UBRs (such as E3s and DUBs) are potential therapeutic targets for cancer treatment, and animal experiments and clinical trials have suggested the therapeutic effects of their corresponding anti-cancer drugs [17, 18]. In addition, several studies have systematically collected parts or all of the UBRs such as UUCD, DUDE-db, iUUCD 2.0, UbiBrowser 1.0 and UbiBrowser 2.0 [2, 10, 19–21]. Although many efforts have been devoted to understanding the physiological functions of UBRs, current comprehensive characterization of UBRs in tissues, developmental stages, cell types and cancer states remains lacking.

Here we systematically analyzed the properties of UBRs across tissues, development and cell types, and comprehensively characterized the molecular perturbation and clinical relevance of UBRs in The Cancer Genome Atlas (TCGA) cohort. The expression pattern of UBRs has heterogeneity across tissues, and the testis is the most distinct. UBRs are dynamically expressed during testicular development, and certain UBRs are specifically expressed in the testis from adolescence to senior. Single-cell transcriptome analysis of the testis revealed that certain UBRs are essential for spermatogenesis. Furthermore, UBRs have widespread genetic alterations and expression perturbations in pan-cancer, and the expression of 79 UBRs was correlated with the activity of 32 cancer hallmark-related pathways. More than 90% of UBRs are associated with patient survival, and some UBRs are potentially valuable markers for prognostic classification. Our work lays the foundation for developing ubiquitination-based anticancer therapeutic strategies.

## Materials and methods

### Collection of UBRs

Our research workflow was shown in Additional file 1: Fig. S1. We collected human and mouse UBRs from recently published database literature[2, 10, 19]. Among them, 877 UBRs in human include 603 writers, 103 erasers, 147 readers and 24 multi; and 335 UBRs in mouse include 218 writers, 43 erasers, 67 readers and 7 multi (Additional file 2: Table S1). In particular, multi represents that UBRs play a variety of roles in the ubiquitination system. For example, *OTUD3* is both a reader and an eraser.

### Collection and processing of transcriptome data

Three human bulk transcriptome datasets were derived from the Genotype-Tissue Expression (GTEx) consortium, Human Protein Atlas (HPA) and FANTOM5 project, respectively [22–24]. The mouse transcriptome dataset was sourced from PRJNA375882 [25]. To identify which UBRs are tissue-specific, we compared the expression levels of UBRs in a specific tissue with the average expression levels of UBRs among all tissues. The residuals (using rlm function) from each UBR to each tissue regression line were calculated, defined as tissue-specific (TS) scores. The UBR was recognized as tissue-specific if its TS score was higher than 2.5 standard deviations [26, 27].

### UBR expression trajectory analysis during testis development

Gene expression data for tissue development of human and mouse was downloaded from the ArrayExpress database by accession numbers E-MTAB-6814 and E-MTAB-6798, separately [28]. The UBRs in testicular development were clustered by fuzzy c-means clustering algorithm, and results were visualized by R packages ComplexHeatmap (version: 2.15.1) and Mfuzz (version: 2.54.0) [29, 30]. Subsequently, Gene Ontology (GO) enrichment analysis was carried out via R packages clusterProfiler (version: 4.2.2) [31] for each cluster.

### Collection and processing of single-cell transcriptome datasets

The preprocessed human testis single-cell transcriptome dataset was downloaded from the GEO database through accession numbers GSE120508 and GSE134144 [32, 33]. The preprocessed mouse testis single-cell transcriptome dataset was obtained under the accession number GSE148032 [34]. K-means clustering analysis was performed based on the average expression profiles of UBRs in different cell populations. To evaluate the reliability of our results across different datasets, we analyzed the expression patterns of UBRs in two additional human or mouse testis studies [35, 36].

In addition, we use the “subsetData” function of the R package Seurat (version: 4.3.0) to extract subsets of specific cell types from the merged Seurat projects [37]. The batch effect among different samples is removed by the harmony algorithm [38]. Subsequent analysis referred to the standard process of Satija lab single cell transcriptome analysis (https://satijalab.org/seurat/index.html). The marker genes for cell type identification in human spermatocytes and sperm cell subclasses were derived from the research of Wang et al. [39].

### Detection of somatic mutation and copy number variation (CNV)

Somatic mutation data covering 10,224 cancer patients across 33 cancer types was obtained from TCGA MAF files (“MC3”) [40] (Additional file 3: Table S2). The CNV dataset was obtained from Genomic Data Commons (GDC) through the R package TCGAbiolinks (version: 2.25.2). TCGAbiolinks is a tool that can query, search, download and prepare relevant GDC data for further analysis [41]. The results are created using R package ComplexHeatmap and maftools (version: 2.10.5) [29, 42].

### Identifying differentially expressed genes in cancer

Gene expression data was downloaded from the TCGA TARGET GTEx cohort in the UCSC XENA project [43]. To increase the reliability of results, we only identified differentially expressed genes in 25 cancer types with no fewer than ten normal samples with Wilcox’s rank sum test. The p-value is adjusted by the Benjamini & Hochberg (BH) method. Genes with adjusted p-values not exceeding 0.01 and fold change not less than twice were regarded as differentially expressed genes.

### Oncogenic pathway activity analysis

To calculate the activity of cancer hallmark-related pathways, we carried out gene set variation analysis (GSVA) using the GSVA (version: 1.42.0) package in R based on the expression profile of UBRs [44]. GSVA is a non-parametric, non-supervised method that can be viewed as a change in the coordinate system of gene expression data from genes to gene sets [44]. The gene set of cancer hallmark-related pathways used in GSVA was extracted from the Molecular Signatures Database through the msigdbr (version: 7.5.1) (https://igordot.github.io/msigdbr/) package in R. To characterize the relationship between UBRs and cancer hallmark-related pathways, we calculated the Pearson correlation coefficient (PCC) between the expression of UBRs and the activity of cancer hallmark-related pathways. Regulator-pathway pairs’ absolute value of PCC higher than 0.5 and adjusted p-value not exceeding 0.01 was deemed significantly correlated.

### Cross-talk analysis among UBRs or oncogenic pathways

The PCC between UBRs was calculated based on the expression profile of UBRs, which were significantly associated with cancer hallmark-related pathways in TCGA TARGET GTEx cohort. Based on the results of GSVA, the PCC among 49 pathways was also calculated and visualized using corrplot (version: 0.92). Subsequently, we constructed a protein-protein interaction (PPI) network between UBRs that are significantly associated with pathways based on the STRING interaction database (https://cn.string-db.org/). Three sub-networks were identified from the PPI network using the Molecular Complex Detection (MCODE) plugin in Cytoscape (https://cytoscape.org/) [45], which are visualized through the software Gephi (https://gephi.org/).

### Clinical correlation analysis of UBRs

The clinical information of cancer patients was gained using R package TCGAbiolinks, and the survival information of patients was obtained from TCGA TARGET GTEx cohort. Patients were divided into high and low groups for each cancer type according to the median expression level of each UBR. Cox proportional risk regression models were used to assess each UBR’s survival risk (Hazard ratio, HR) in various cancer types. Consensus clustering analysis of patients was performed using ConseusClusterPlus (version: 1.58.0) for nine cancer types based on the expression of hub UBRs [46]. Except for clusterAlg = “pam”, distance = “spearman” and reps = 2000 bootstraps, all other parameters are default. The log-rank test of Kaplan-Meier survival was used to confirm whether there was a difference in survival rate between the two groups, and the survival curve was built with the survminer (version: 0.4.9) package.

### Drug sensitivity analysis

To elucidate the relationship between hub gene expression and drug sensitivity and resistance, we downloaded transcriptome data and drug sensitivity data (IC50) of human cancer cell lines from the CellMiner database [47]. From 24,620 compounds, 860 drugs or compounds that entered clinical trials (546) or FDA (US Food and Drug Administration) approval (314) were screened for further analysis. Subsequently, we calculated the PCC between the IC50 values of 860 drugs or compounds and the expression levels of hub genes. Genes and drugs or compounds with p-values less than 0.01 were retained.

### Statistical analysis

All the above statistical analyses were performed in R (version: 4.1.1) software. Except for special instructions, a p-value no more than 0.05 was used as the threshold of statistical significance. The statistical methods used are described fully in the corresponding sections above.

## Results

### Heterogeneity in expression patterns of UBRs across tissues

**Fig. 1:**
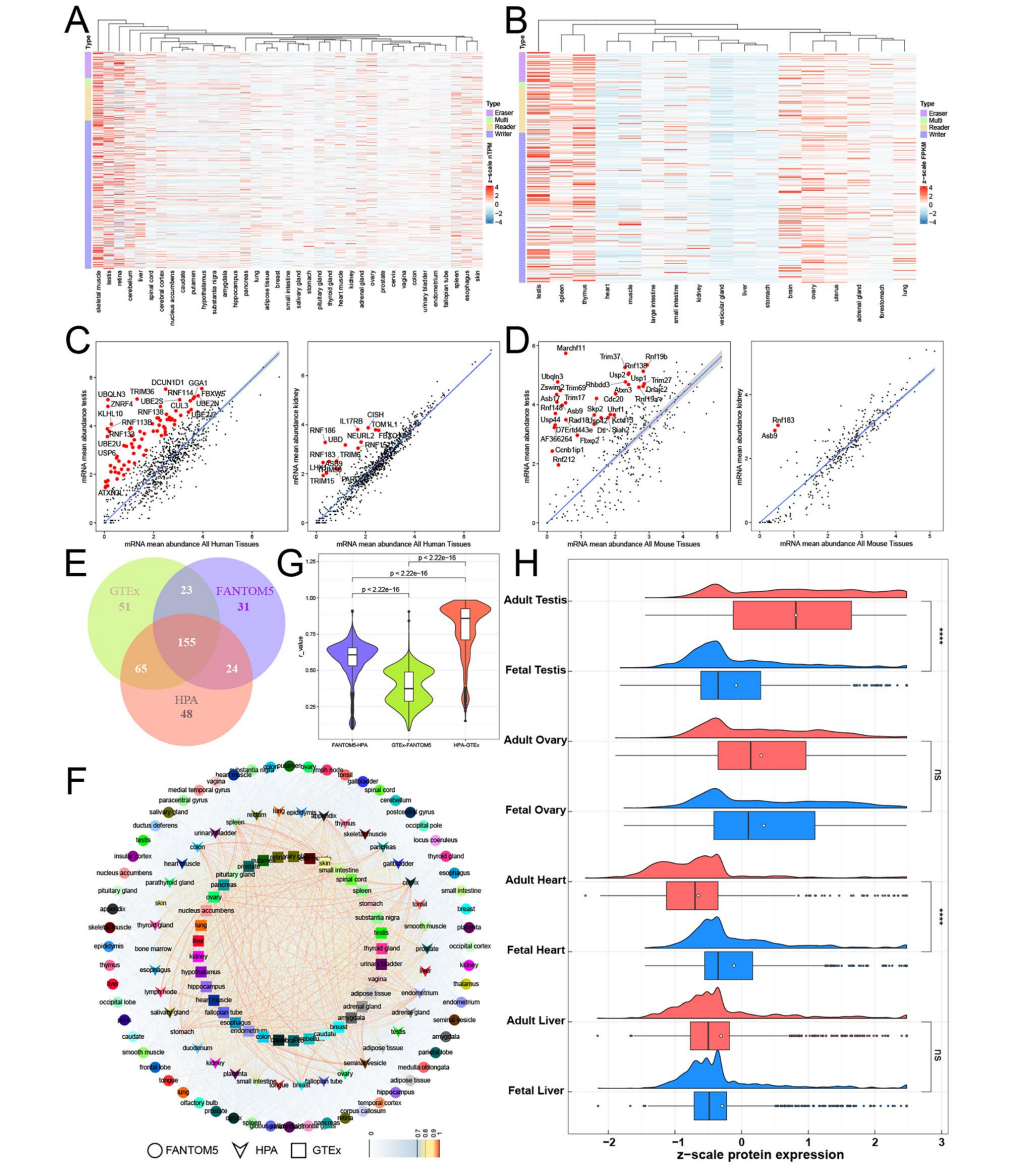
Expression pattern of UBRs across tissues. (**A-B**) Heatmap of UBRs expression levels. (**A**) human, (**B**) mouse. (**C-D**) Scatter plots that visualize the tissue-specific UBRs. (**C**) human, (**D**) mouse. Tissue-specific UBRs are labeled with red. (**E**) Venn diagrams of tissue-enriched UBRs. (**F**) Network showing Pearson correlations among tissues in three independent datasets. Internal links display the PCC. (**G**) Violin plots showing the distribution of PCCs among the tissues in the three datasets. (**H**) Raincloud shows the protein expression levels of UBRs in fetal and adult tissues

To determine the expression pattern of UBRs across tissues in human and mouse, we examined the heterogeneity of UBRs’ expression patterns using transcriptome datasets [22, 25]. UBRs had higher expression levels in skeletal muscle, testis, and retina than other tissues in human (Fig. 1A). In addition, UBRs also had higher expression levels in testis, spleen, and thymus than other tissues in mouse (Fig. 1B). To further explore the characteristics of UBRs across tissues, we calculated the TS scores of each UBR in each tissue. Testis is the most special tissue in terms of UBRs expression patterns (Fig. 1C-D; Additional file 4: Table S3). Although the average expression level of UBRs in human skeletal muscle was higher than that in testis, the number of tissue enrichment genes in skeletal muscle was lower than that in testis (Additional file 1: Fig. S2A). This was not only due to the quasi-exclusively expression of several UBRs in the testis, but also due to the significantly increased expression levels of several UBRs in the testis (Additional file 1: Fig. S2B). By contrast, other tissues such as stomach and kidney showed less tissue-enriched UBRs (Fig. 1C-D).

Two other transcriptome datasets were collected to verify universal expression pattern of UBRs in human tissues [23, 24]. As expected, UBRs had similar expression patterns in three datasets (Additional file 1: Fig. S2C-D; Additional file 4: Table S3) and the tissue-enriched UBRs identified in the three datasets were highly overlapped (Fig. 1E). Next, we calculated the PCC among tissues in three datasets based on the expression of UBRs. The results observed high correlations of the same tissues in three datasets, implying that UBRs were conserved across datasets (Fig. 1F). The tissues of GTEx and HPA datasets have a higher correlation, which may be related to the heterogeneity of datasets (Fig. 1G).

Then we wondered whether the tissue-specific expression patterns of UBRs could also be observed at the proteome level. We found that testis’ UBRs displayed the most distinctive protein expression patterns among the 18 tissues [48] (Additional file 1: Fig. S3A). By contrast, other tissues showed few tissue-enriched UBRs (Additional file 1: Fig. S3B-C; Additional file 5: Table S4). Besides that, UBRs are differentially expressed in several fetal and adult tissues (Fig. 1H), which implies that UBRs may play an essential role in specific tissue development.

### Dynamic expression of UBRs in tissue development

Transcriptome datasets covering multiple developmental stages were used to explore the expression pattern of UBRs [28]. We found UBRs were highly dynamically expressed during tissue development and the expression pattern of many UBRs changes postnatally (Additional file 1: Fig. S4). Notably, some UBRs have a specifically high expression level from puberty to senior in testis, suggesting that they involved in male reproduction.

**Fig. 2:**
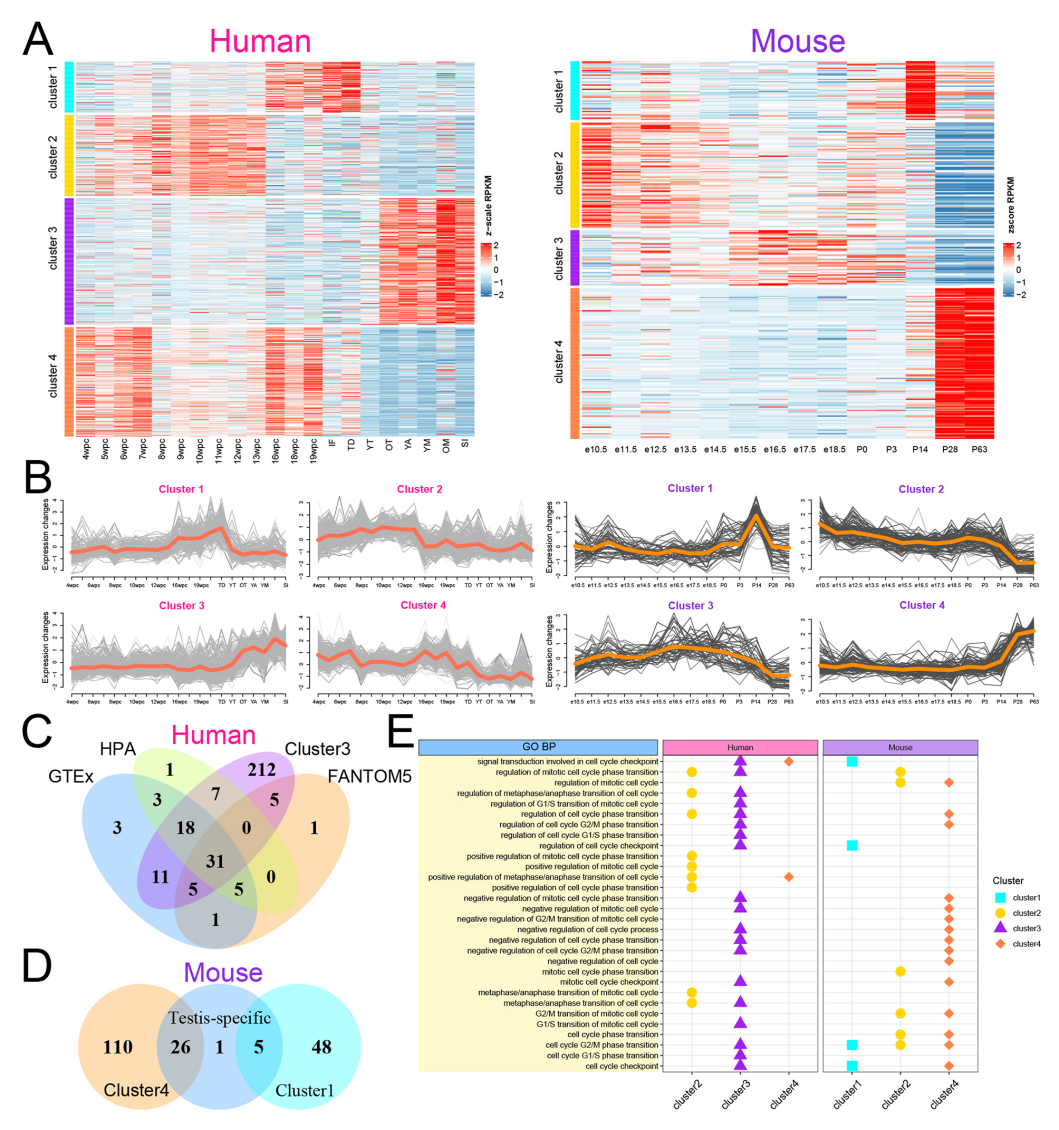
Expression patterns of UBRs during testis development. (**A**) Expression heatmap of UBRs during testis development. Left, human; right, mouse. TF: infant; TD: toddler; YT: youngTeenager; YA: youngAdult; YM: youngMidAge; OM: olderMidAge. (**B**) The temporal clustering analysis of UBRs expression in testis development. Left, human; right, mouse. (**C-D**) Venn diagram of genes in specific clusters and testis-specific genes. (**E**) Comparison of cell cycle-related GO terms in different clusters. The same clusters in human and mouse have the same color and shape

Subsequently, we analyzed the dynamic changes in the expression profile of UBRs during testis development by soft clustering with Mfuzz [30]. UBRs were classified into four clusters in both human and mouse testis (Fig. 2A-B; Additional file 6: Table S5). Interestingly, UBRs in cluster 3 of human testes were highly expressed from the OT (oldTeenager) to SI (Senior) stages, suggesting that they are closely associated with male reproduction (Fig. 2B). UBRs in cluster 4 of mouse testis have higher expression levels in adulthood (P28 to P63), suggesting that they have a similar role to the UBRs in human testis cluster 3 (Fig. 2B). Furthermore, UBRs in human cluster 3 highly overlapped with those tissue-enriched in testis, and similar results were found in mouse (Fig. 2C-D), implicating that some UBRs play critical roles in reproduction-related processes. Then, we analyzed and compared the GO terms of UBRs in each cluster. UBRs in human cluster 3 and mouse cluster 4 are significantly enriched in cell cycle-related biological processes, while UBRs in other clusters are less enriched in cell cycle-related biological processes (Fig. 2E). It implied UBRs in human cluster 3 and mouse cluster 4 may play a vital role in spermatogenesis.

### UBRs are essential for spermatogenesis

**Fig. 3:**
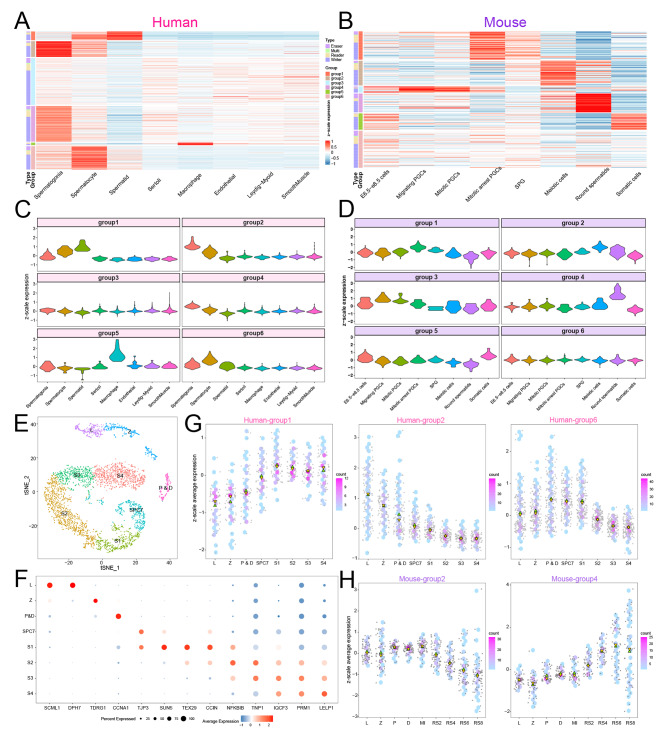
Expression pattern of UBRs in spermatogenesis. (**A-B**) Heatmap of UBRs expression across cell types. (**C-D**) Violin diagram of UBR expression pattern in each group of testis. (**C**) human, (**D**) mouse. (**E**) The tSNE diagram of spermatocytes and sperm subclasses in human testis. L: leptotene; Z: zygotene; P & D: pachytene and diplotene; SPC7: spermatocyte 7; S: spermatid. (**F**) Expression bubble maps of marker genes. (**G-H**) Expression scatters plot of UBRs across cell types. (**G**) Human, (**H**) Mouse. L: leptotene; Z: zygotene; P: pachytene; D: diplotene; MI: metaphase I; RS: round spermatid

To comprehensively identify which stages of sperm formation and maturation the UBRs are involved in, we analyzed the UBRs’ expression patterns in different cell types using k-means clustering, which could be classified into six types in both human and mouse testis (Fig. 3A-D; Additional file 7: Table S6). In human, group 1, 2, 4 and 6 were highly expressed in germ cells, including spermatogonia (SPG), spermatocytes (SPC) and spermatids (S); group 5 was highly expressed in macrophages; while group 3 was expressed in almost all cell types. In mouse, group 1 and 3 were highly expressed in primordial germ cells (PGCs) and SPG; group 2 and 4 were highly expressed in meiotic cells and round spermatids (RS); group 5 was highly expressed in somatic cells, while group 6 expressed in nearly all cell types. Besides that, we observed similar expression patterns in other human and mouse testis datasets (Additional file 1: Fig. S5).

To further explore the role of UBRs in meiosis and sperm maturation, spermatocytes and spermatids were classified into more specific cell types [34, 39] (Fig. 3E-F). UBRs in human group 1 were highly expressed from SPC7 (spermatocyte 7) to S4 stages, suggesting a role in late meiosis and spermatids maturation to morphogenesis. The UBRs in human group 2 functioned at the mitosis and meiosis (leptotene (L) to diplotene (D)), while UBRs in human group 6 functioned in the meiosis and spermatids S1 stages (Fig. 3G). Furthermore, UBRs in mouse group 2 functioned at all stages of meiosis and the RS2 phase of spermatids, whereas most UBRs in mouse group 4 functioned in the RS4 to RS8 phase of spermatids (Fig. 3H). Our analysis implied that ubiquitination homeostasis is critical for normal spermatogenesis.

### Genetic alterations and dysregulation of UBRs in pan-cancer

The deregulation of ubiquitin pathways leads to the development of human diseases [14, 49]. Aberrant ubiquitination is more commonly caused by mutation or abnormal expression of genes that encode E3s or DUBs [12]. The FDA has approved three small-molecule drugs (thalidomide, lenalidomide and pomalidomide) that target E3s [50, 51]. Therefore, a systematic understanding of the genetic alterations and dysregulation of UBRs in cancer can provide new insights into targeted anti-cancer therapies.

**Fig. 4:**
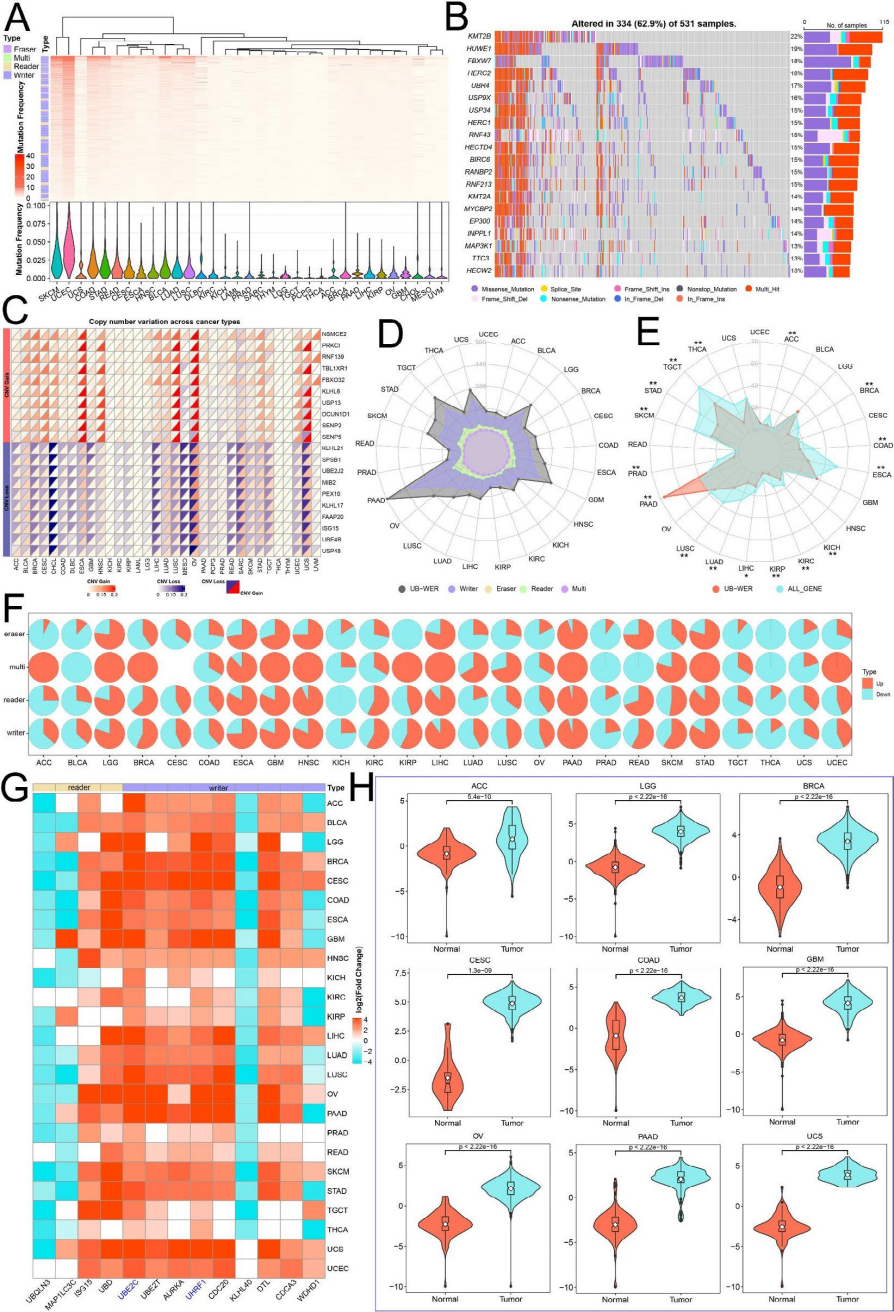
Genetic alterations and expression dysregulation of UBRs in pan-cancer. (**A**) Somatic mutation frequency of UBRs across different cancers. (**B**) The top 20 UBRs with the highest mutation frequency in UCEC. (**C**) Heatmap of CNV alteration frequencies across different cancers for partial UBRs. (**D**) Quantitative distribution of dysregulated UBRs in 25 cancer types. (**E**) The percentage distribution of dysregulated genes in UBRs and in all genes. (**F**) Distribution of dysregulated UBRs in the same functional category in 25 cancer types. (**G**) Heatmap showing the expression changes of 13 UBRs. (**H**) Expression levels of *UHRF1* in normal and cancer samples

Then, we assessed the frequency of non-silent somatic mutations and CNVs of UBRs in pan-cancer. Although the overall mean mutation frequency of UBRs is low, with a span of 0.02–4.9% (Fig. 4A; Additional file 8: Table S7), UBRs have a high global mutation burden in specific cancer types (such as UCEC and SKCM). In 531 UCEC patients, almost all had mutations in UBRs. Among them, the mutation frequency of *KMT2B* was the highest (22%), while *FBXO17* and *USP9Y* did not display any mutations (Fig. 4B; Additional file 8: Table S7). On the contrary, UBRs in several cancer types (such as TGCT, THCA and PCPG) showed fewer mutations than other cancers (Fig. 4A). Subsequently, we examined the CNVs of UBRs. The results showed UBRs have extensive CNV gain and CNV loss in pan-cancer (Additional file 1: Fig. S6A; Additional file 8: Table S7). *NSMCE2* and *PRKCI* have a wide frequency of CNV gain, while *KLHL21* and *SPSB1* have a broad frequency of CNV loss (Fig. 4C). Compared with other cancer types, UBRs have higher CNV gain and CNV loss frequencies in OV (Additional file 1: Fig. S6C). The frequency of CNV gain and CNV loss of UBRs significantly differed among 28 cancer types (Additional file 1: Fig. S6B).

Then, we found that UBRs had extensively dysregulated across cancers, and the number of dysregulated genes in UBRs is closely related to cancer types, ranging from 84 to 552 (Fig. 4D). Therefore, we wanted to know whether UBRs are more prone to expression perturbations than other genes. The results showed that the proportion of dysregulated genes in UBRs is significantly different from that of all genes in the 16 cancer types (Fig. 4E). Interestingly, the proportion of deregulated genes in writers and erasers was different across multiple cancer types (Fig. 4F), implicating a widespread imbalance of ubiquitin regulatory networks. In addition, 13 UBRs were deregulated in 20 or more cancer types (Fig. 4G), of which *UHRF1* (Fig. 4H) and *UBE2C* were both deregulated in 25 cancer types.

### Oncogenic pathways regulated by UBRs

**Fig. 5:**
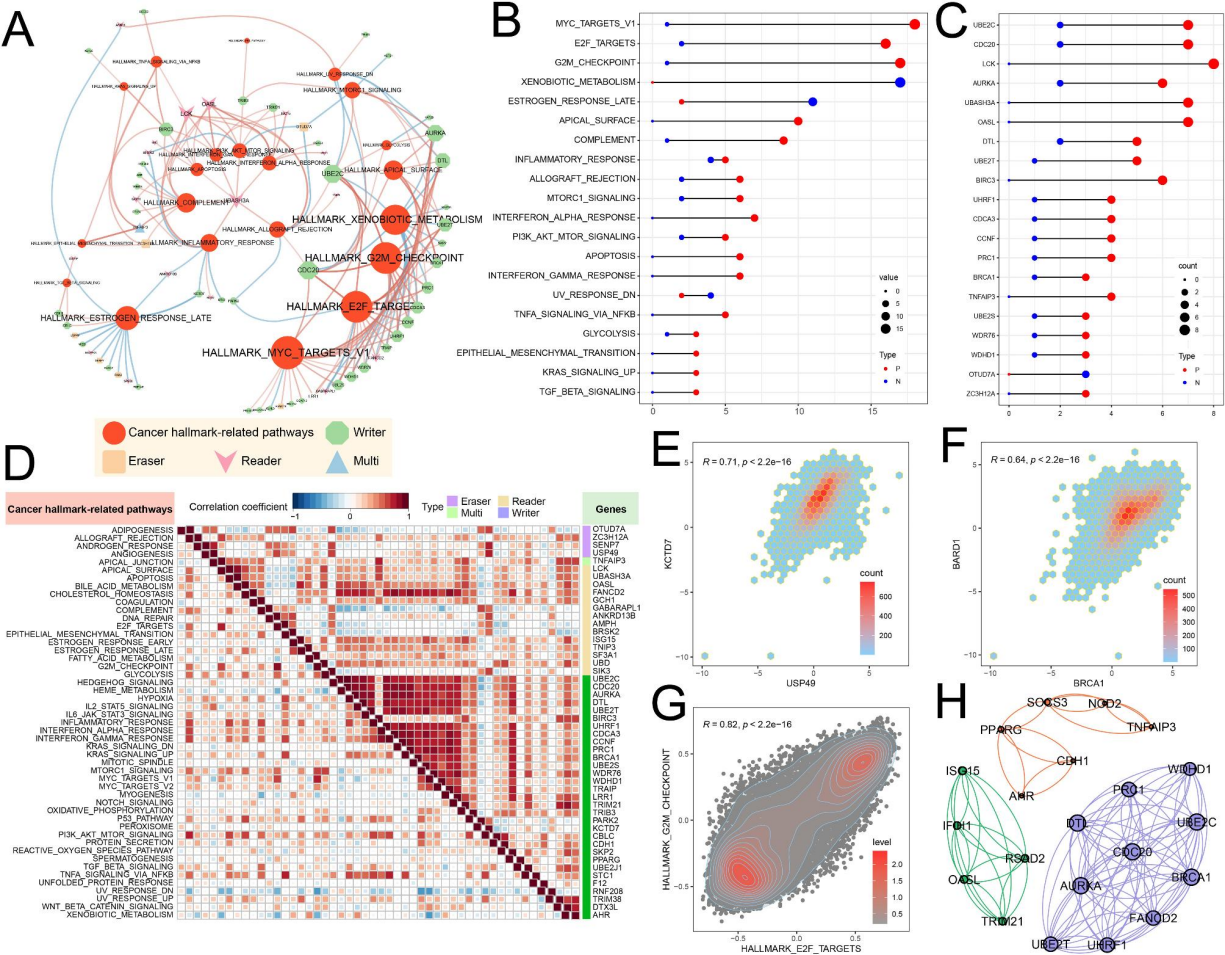
Analysis between UBRs and cancer hallmark-related pathways. (**A**) Correlation network between UBRs and pathways. The red line means a positive correlation and the blue line means a negative correlation. (**B**) Quantitative distribution of UBRs that were significantly correlated with specific pathways. (**C**) Quantitative distribution of pathways that were significantly correlated with specific UBRs. (**D**) Correlation between pathways or partial UBRs. Bottom left, correlation between pathways; top right, correlation between UBRs. (**E**) Correlation between *USP49* and *KCTD7*. (**F**) Correlation between *BRCA1* and *BARD1*. (**G**) Correlation between E2F targets pathway and G2M checkpoint pathway. (**H**) Subnetworks identified from the 79 UBRs’ PPI networks. Blue represents the subnetwork 1

To further explore the molecular mechanism and biological function of UBRs in pan-cancer, we analyzed the correlation between the expression of UBRs and the activity of cancer hallmark-related pathways. We found the expression of 79 UBRs correlated with the activation or inhibition of 32 oncogenic pathways (Fig. 5A; Additional file 9: Table S8). 21 oncogenic pathways’ activity such as MYC targets V1 and G2M checkpoint, correlates with the expression of multiple UBRs (Fig. 5B). By contrast, 11 oncogenic pathways’ activity only correlates with the expression of one UBR. Interestingly, the expression of the majority of UBRs was positively correlated with the activity of cancer hallmark-related pathways (Fig. 5C; Additional file 9: Table S8). Furthermore, different functional classes of UBRs were associated with different cancer pathway alterations, suggesting that the same functional class of UBRs has different functional effects.

Then we investigated expression correlations between UBRs and the correlations between cancer hallmark-related pathways. There are highly correlated expression patterns among UBRs, regardless of whether they belong to the same functional class (Fig. 5D). For instance, the reader *USP49* was significantly correlated with the writer *KCTD7* (Fig. 5E). Notably, genes in the same protein complex have higher correlations, such as *BRCA1* and *BARD1* (Fig. 5F). Furthermore, there are widespread correlations between cancer hallmark-related pathways (Fig. 5D). For example, E2F targets pathway was highly correlated with G2M checkpoint pathway (Fig. 5G). Subsequently, we constructed the PPI network of 79 UBRs based on the STRING database, and the results showed extensive interactions between them. We obtained three sub-networks by the plugin MCODE [45], where the genes in subnetwork 1 are considered as hub genes of the whole network (Fig. 5H).

### Clinical relevance of UBRs in pan-cancer

**Fig. 6:**
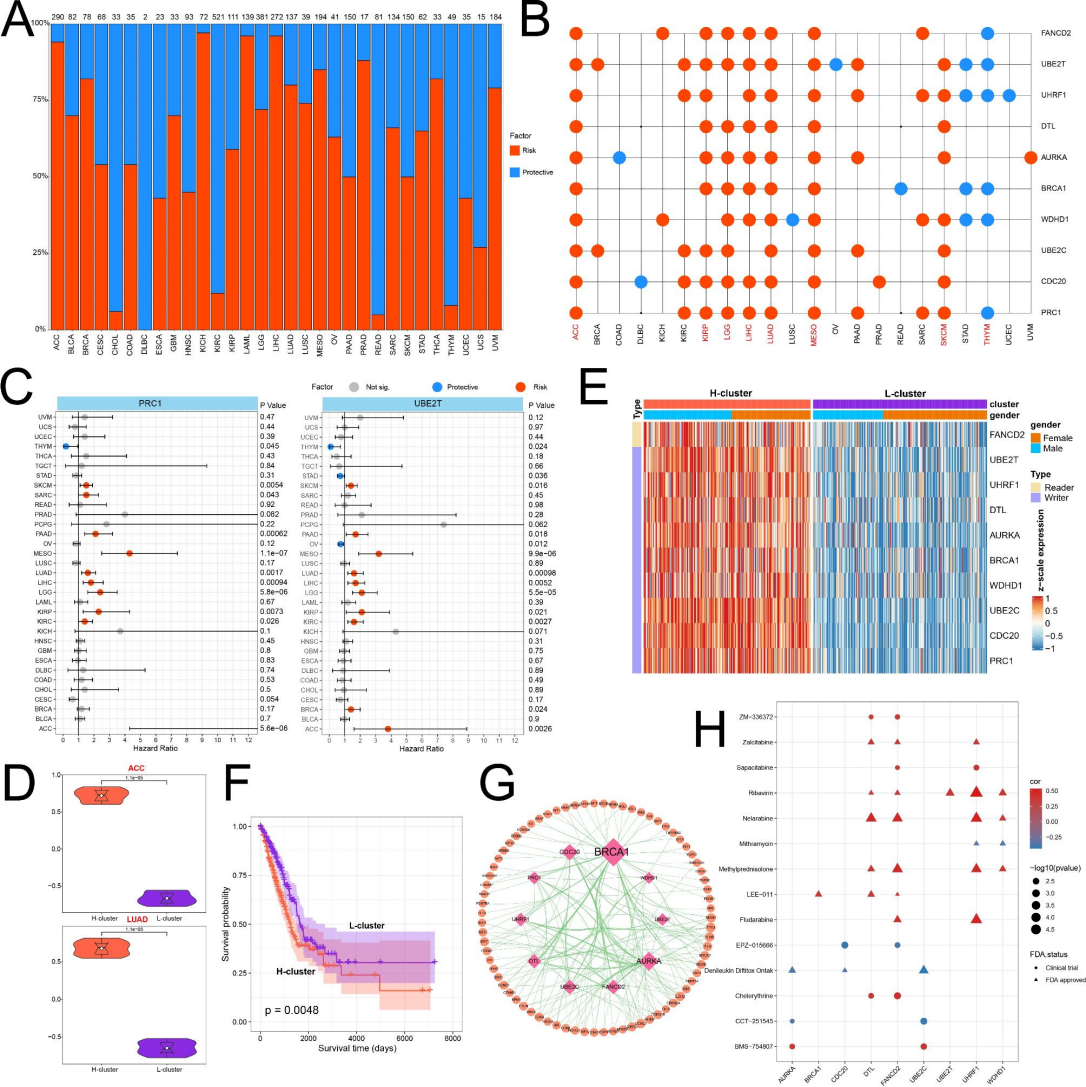
Clinical correlation analysis of UBRs in pan-cancer. (**A**) The quantitative distribution of UBRs affecting patient survival. (**B**) Impact of hub genes on patient survival. (**C**) HRs distribution for specific genes. (**D**) Violin plot showing average expression of hub genes in different clusters. (**E**) The expression heatmap of hub gene in LUAD. (**F**) Kaplan-Meier survival plots for LUAD in different clusters. (**G**) PPI network between hub genes and clinically actionable genes. (**H**) Drug sensitivity analysis of hub genes. Color represents the correlation between gene expression and drug IC50. The size of the point is inversely related to the size of the p-value. The shape represents the state of the drug, the circle represents the drug in the clinical trial stage, and the triangle represents the drug that has been approved by the FDA

We further excavated UBRs’ prognostic relevance using TCGA clinical data. The majority of UBRs (825/877) were associated with patient survival in cancer, more than 90% of which functioning in multiple cancer types (Fig. 6A; Additional file 10: Table S9). Notably, the number (ranging from 0 to 521) of UBRs associated with patient overall survival was strongly correlated with cancer types. Among them, KIRC had the highest number of UBRs associated with patient survival, whereas TCGT and PCPG had the lowest number. Subsequently, we found hub genes can affect the survival and prognosis of patients in 22 cancer types. Among them, the high expression of hub genes in 8 cancer types is beneficial to the overall survival of patients, while the high expression of hub genes in 14 cancer types is adverse to the overall survival of patients (Fig. 6B). Especially, several hub genes exhibit carcinogenic properties (Fig. 6C), and high expression of these genes is associated with worse survival.

Moreover, we focused on eight cancer types, including ACC, KIRP, LGG, LIHC, LUAD, MESO, SKCM and THYM, in which more than half of the hub genes affected patient overall survival. Based on the expression of hub genes, consensus matrix showed that the best classification across the eight cancer types was all 2 clusters (Additional file 1: Fig. S7). Especially, there was a significant difference in the mean expression of hub genes between different clusters of the same cancer type (Fig. 6D, p < 0.01), so we divided the clusters into high expression clusters (H-cluster) and low expression clusters (L-cluster) to compare the overall survival of patients in different clusters (Additional file 1: Fig. S8-9). The results revealed that in THYM, the prognosis of patients in the H-cluster was not significantly different from that in the L-cluster, while patients in L-cluster had a better prognosis among the other seven cancer types (Fig. 6E-F; Additional file 1: Fig. S10). To better understand the clinical implications of hub genes, we explored the correlation between hub genes and 123 clinically actionable genes [52], and observed frequent interactions between them (Fig. 6G). In addition, we calculated the PCC between drug sensitivity (IC50) and gene expression profile data in cancer cell lines [47]. The results showed that the expression of 10 hub genes was significantly correlated with the sensitivity of 64 clinical trials or FDA-approved drugs, of which 14 drugs were associated with multiple hub genes (Fig. 6H; Additional file 11: Table S10). For example, the sensitivity of FDA-approved drugs such as Zalcitabine, Ribavirin and Methylprednisolone was positively correlated with the expression of *DTL*, *FANCD2* and *UHRF1*. The sensitivity of EPZ − 015666 in clinical trials was negatively correlated with the expression of *CDC20* and *FANCD2*. Our results suggest that the expression of hub genes may mediate drug resistance to targeted drug therapy and can provide new insights into the development of anticancer drugs.

## Discussion

Ubiquitination controls almost all cellular processes [53, 54]. Targeted at components of the ubiquitination machinery has emerged as an effective therapeutic intervention strategy. UBRs are involved in writing, erasing and reading of ubiquitination, but their role in carcinogenesis and potential as therapeutic targets have not been characterized to a great degree. To bridge this gap, here we systematically explored the expression patterns and signatures of UBRs across tissues, developmental periods, cell types and cancers.

Spermatogenesis depends on the balance between ubiquitination and deubiquitination [55, 56]. Our study revealed that the expression pattern of UBRs across tissues is highly heterogeneous. Notably, the expression pattern of UBRs in the testis is the most distinct. UBRs are selectively expressed during testicular development, and certain UBRs are specifically expressed from puberty to senior, implying that they are closely associated with male reproduction. For instance, *UBE2J1* is required for the elongation phase of spermatids, and spermatids from *Ube2j1*-knockout mice are thought to be defective in the dislocation step of endoplasmic reticulum quality control [57]. *CUL4A* plays an important role in DNA replication, chromatin condensation and cell cycle. In *Cul4A*-deficient mice, there is a decrease in testicular weight and an increase in abnormal multinucleated and apoptotic germ cells [58]. In addition, analysis of single-cell transcriptome data from human and mouse testis revealed that UBRs are selectively expressed during spermatogenesis and are critical for normal mitosis, meiosis, sperm maturation and deformation during spermatogenesis. For example, *UHRF1* is a critical regulator in DNA methylation retention and histone modification, which is essential for spermatogenesis. Conditional deficiency of *Uhrf1* in differentiated spermatogonia results in meiotic defects and infertility [59]. Mutations in *RNF126* and *RNF12* cause Gordon Holmes syndrome and X-linked intellectual disability, respectively. Patients suffering from either of these two diseases have low sex hormone levels and abnormally small testes [13].

Ubiquitination regulation is multifaceted and plays a role in ensuring cell homeostasis and life activities. When ubiquitination regulatory mechanisms change, the altered biological processes may later induce multiple cancers [16]. We comprehensively analyzed the expression perturbations and genetic changes of UBRs in pan-cancer. The expression of 79 UBRs was significantly correlated with the activity of 32 cancer marker-related pathways. The majority of UBRs could affect the survival of patients, and certain of them had a good prognostic classification for patients. Our analysis will contribute new insights into drug development targeting UBRs. Notably, more and more studies have demonstrated that UBRs are expected to provide new strategies for cancer treatment, especially E3s and DUBs [13, 17, 18, 60, 61]. At present, many small molecule inhibitors targeted at UBRs have been developed, such as MLN7243 and MLN4924 (targeting E1s), 4,5-dihydroimidazoline (targeting E3s), broad-spectrum inhibitor NSC632839 and specific inhibitor Pimozide (targeting DUBs) [16].

## Conclusion

Our study systematically analyzed the molecular characteristics and potential functions of UBRs across tissues and revealed that they are essential for spermatogenesis. Subsequently, we analyzed the genetic alterations, expression perturbations, carcinogenic pathways and clinical relevance of UBRs in pan-cancer. Our work emphasizes the importance of UBRs in spermatogenesis and pan-cancer, providing new perspectives on the pathogenesis and treatment of infertility and cancer.

Our work aims to systematically study the characteristics of UBRs in spermatogenesis and pan-cancer and to further perform a limited analysis of the subset of inference functions. Therefore, we cannot conduct an in-depth analysis of individual genes or specific cancer types. We only selected hub genes based on PPI networks, ignoring the heterogeneity among different cancer types, which can be explored from more perspectives in future studies. Taken together, Our work can provide guidance for the treatment of infertility and the development of cancer-targeted drugs.

### Electronic supplementary material

Below is the link to the electronic supplementary material.

Supplementary Material 1

Supplementary Material 2

Supplementary Material 3

Supplementary Material 4

Supplementary Material 5

Supplementary Material 6

Supplementary Material 7

Supplementary Material 8

Supplementary Material 9

Supplementary Material 10

Supplementary Material 11

## Data Availability

All datasets in this study are available from public online repositories, and the repository names and accession numbers are discoverable in the manuscript or supplementary materials. In short, the human bulk transcriptome dataset was derived from the GTEx, HPA and FANTOM5 project; the proteome dataset was derived from the human proteome map (http://www.humanproteomemap.org/); human development transcriptome data was derived from the ArrayExpress database (accession numbers: E-MTAB-6814); the single-cell transcriptome dataset of human testis was derived from the GEO database with accession numbers GSE142585, GSE120508 and GSE134144, respectively. The mouse transcriptome dataset was obtained from multiple databases through the accession numbers of PRJNA375882, E-MTAB-6798, GSE148032 and GSE112393. The main R scripts used in this work are available on GitHub (https://github.com/DYL-LiaoLab/Comprehensive_analysis_of_UBRs).

## Abbreviations

UBRs: Ubiquitination regulators
GTEx: Genotype-Tissue Expression consortium
HPA: Human Protein Atlas
TS scores: Tissue-specific scores
GO: Gene Ontology
GSVA: Gene set variation analysis
CNV: copy number variation
PCC: Pearson correlation coefficient
MCODE: Molecular Complex Detection
ACC: Adrenocortical carcinoma
LAML: Acute myeloid leukemia
BLCA: Bladder urothelial carcinoma
BRCA: Breast invasive carcinoma
LGG: Brain lower grade glioma
CESC: Cervical squamous cell carcinoma and endocervical adenocarcinoma
CHOL: Cholangiocarcinoma
COAD: Colon adenocarcinoma
DLBC: Lymphoid neoplasm diffuse large B-cell lymphoma
ESCA: Esophageal carcinoma
GBM: Glioblastoma multiforme
HNSC: Head & neck squamous cell carcinoma
KICH: Kidney chromophobe
KIRC: Kidney renal clear cell carcinoma
KIRP: Kidney renal papillary cell carcinoma
LIHC: Liver hepatocellular carcinoma
LUAD: Lung adenocarcinoma
LUSC: Lung squamous cell carcinoma
MESO: Mesothelioma
OV: Ovarian serous cystadenocarcinoma
PAAD: Pancreatic adenocarcinoma
PCPG: Pheochromocytoma and paraganglioma
PRAD: Prostate adenocarcinoma
READ: Rectum adenocarcinoma
SARC: Sarcoma
SKCM: Skin cutaneous melanoma
STAD: Stomach adenocarcinoma
TGCT: Testicular germ cell tumor
THYM: Thymoma
THCA: Thyroid carcinoma
UCS: Uterine Carcinosarcoma
UCEC: Uterine corpus endometrioid carcinoma
UVM: Uveal melanoma
