# Supplementary material for: Integrated analysis of the ubiquitination mechanism reveals the specific signatures of tissue and cancer
Source: BMC Genomics. 2023 Sep 4;24:523. doi: 10.1186/s12864-023-09583-z (PMC10478310; doi:10.1186/s12864-023-09583-z)
Supplement: Supplementary file 1 — Supplementary Material 1 [file 12864_2023_9583_MOESM1_ESM.docx]

**Integrated analysis of the ubiquitination mechanism reveals the specific signatures of tissue and cancer**

Deyu Long^1,3^, Ruiqi Zhang^1^, Changjian Du^1^, Jiapei Tong^2^, Yu Ni^1^, Yaqi Zhou^1^, Yongchun Zuo^3*^, Mingzhi Liao^1*^

^1^Center of Bioinformatics, College of Life Sciences, Northwest A&F University, Yangling, Shaanxi 712100, China.

^2^College of Information Engineering, Northwest A&F University, Yangling, Shaanxi, China.

^3^State Key Laboratory of Reproductive Regulation and Breeding of Grassland Livestock, College of Life Sciences, Inner Mongolia University, Hohhot 010070, China.

^*^Corresponding authors. Yongchun Zuo, Email: [yczuo@imu.edu.cn](mailto:yczuo@imu.edu.cn); Mingzhi Liao, Email: [liaomingzhi83@163.com](mailto:liaomingzhi83@163.com).


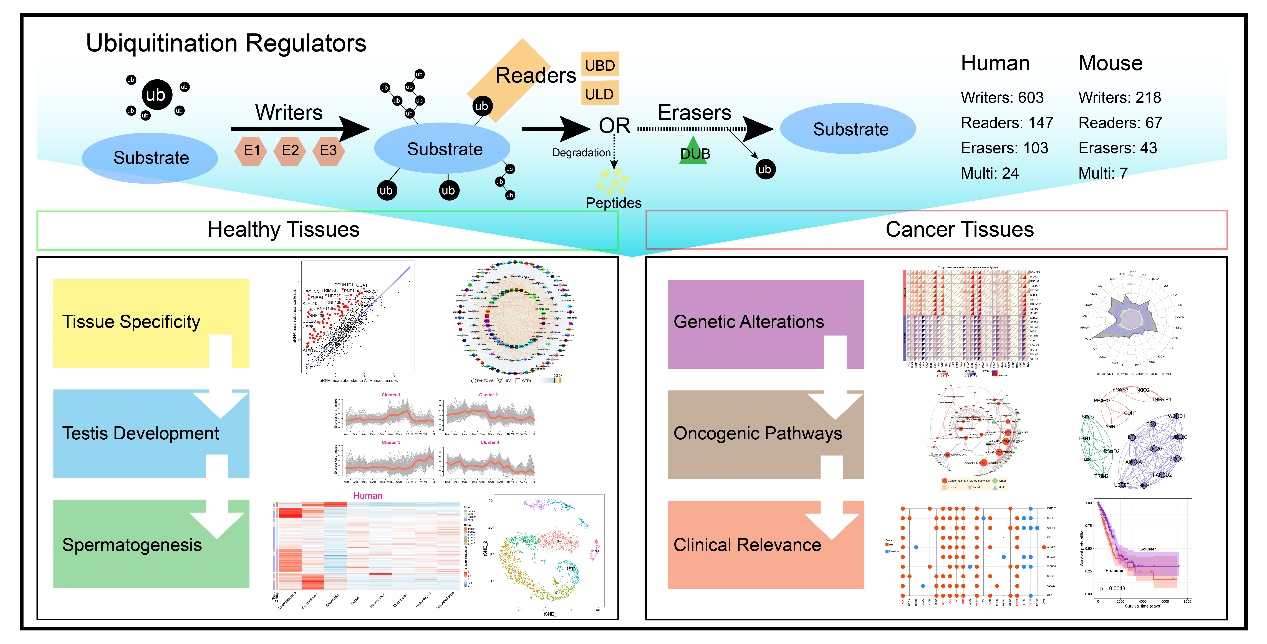


Supplementary Fig. S1. An overview of the study of UBRs across tissues and cancers.


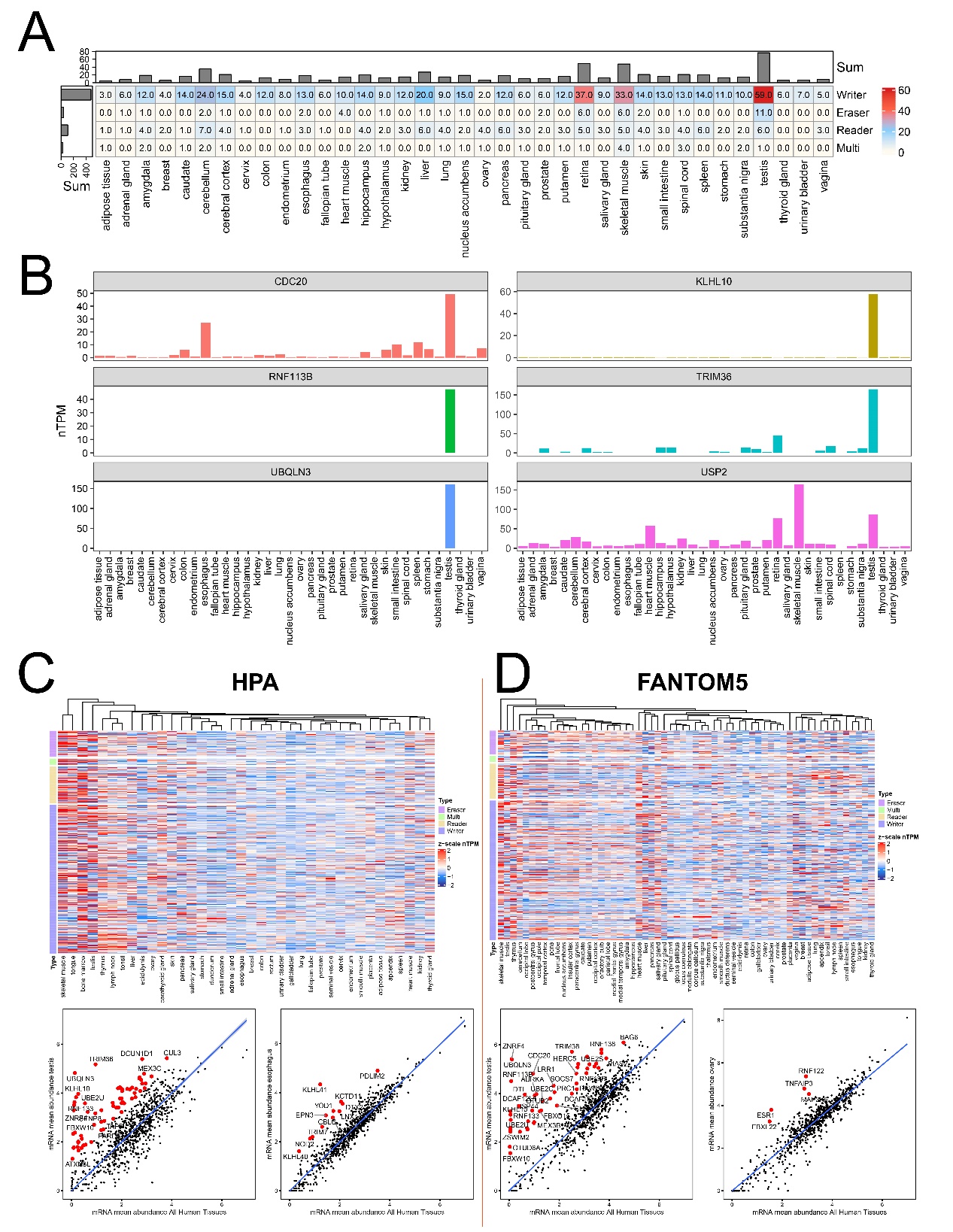


Supplementary Fig. S2. Expression patterns of UBRs across tissues. (**A**) The distribution of tissue-enriched genes in the GTEx dataset. (**B**) Expression patterns of six testis-enriched genes in the GTEx dataset. (**C**-**D**) The expression heatmap of UBRs and the scatter plot of UBRs tissue-enriched analysis.


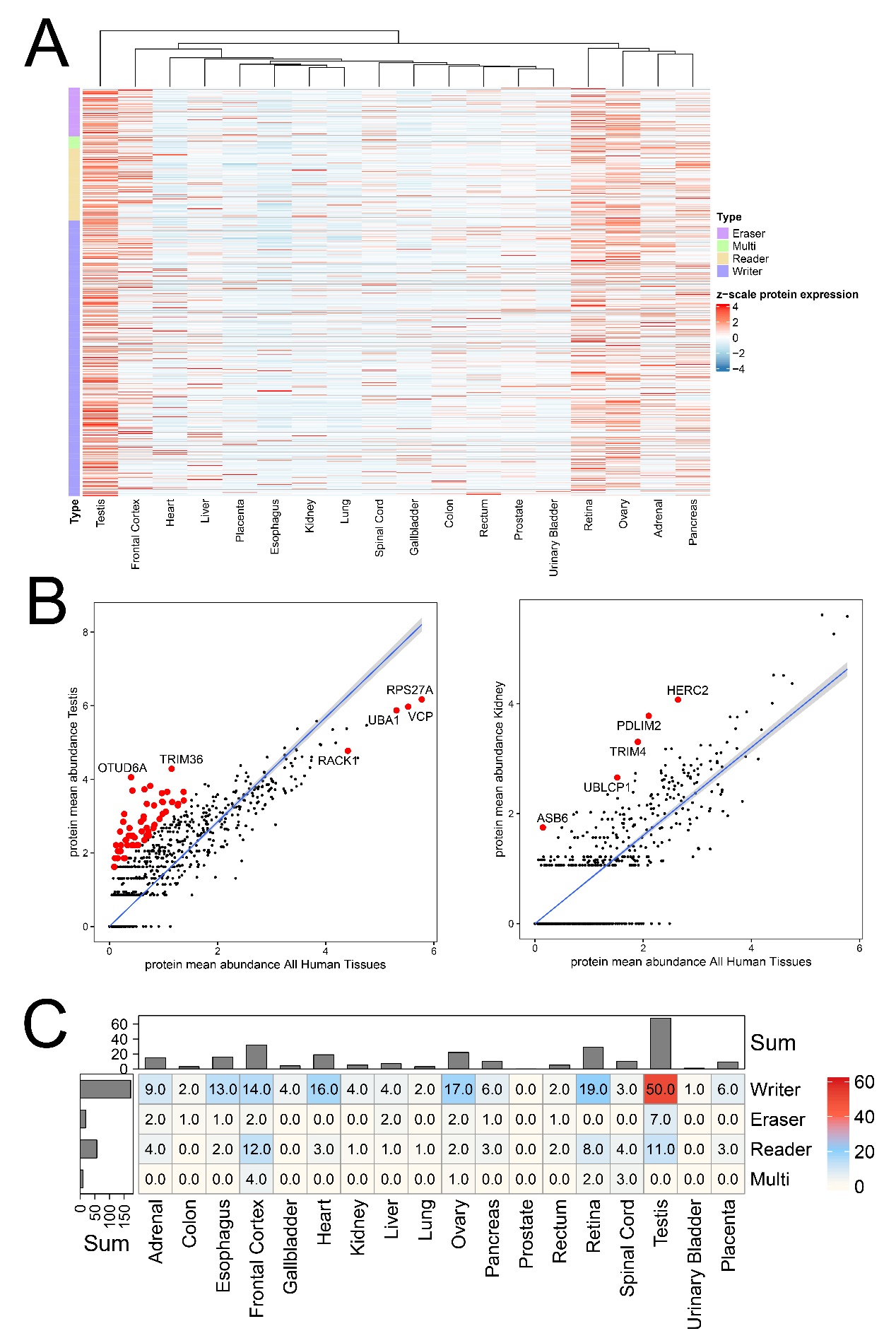


Supplementary Fig. S3. Cross-tissue expression patterns of UBRs at the protein level. (**A**) Heatmap of UBRs expression. (**B**) Scatter plot of UBR tissue-enriched analysis. (**C**) Quantity distribution of tissue-enriched UBRs.


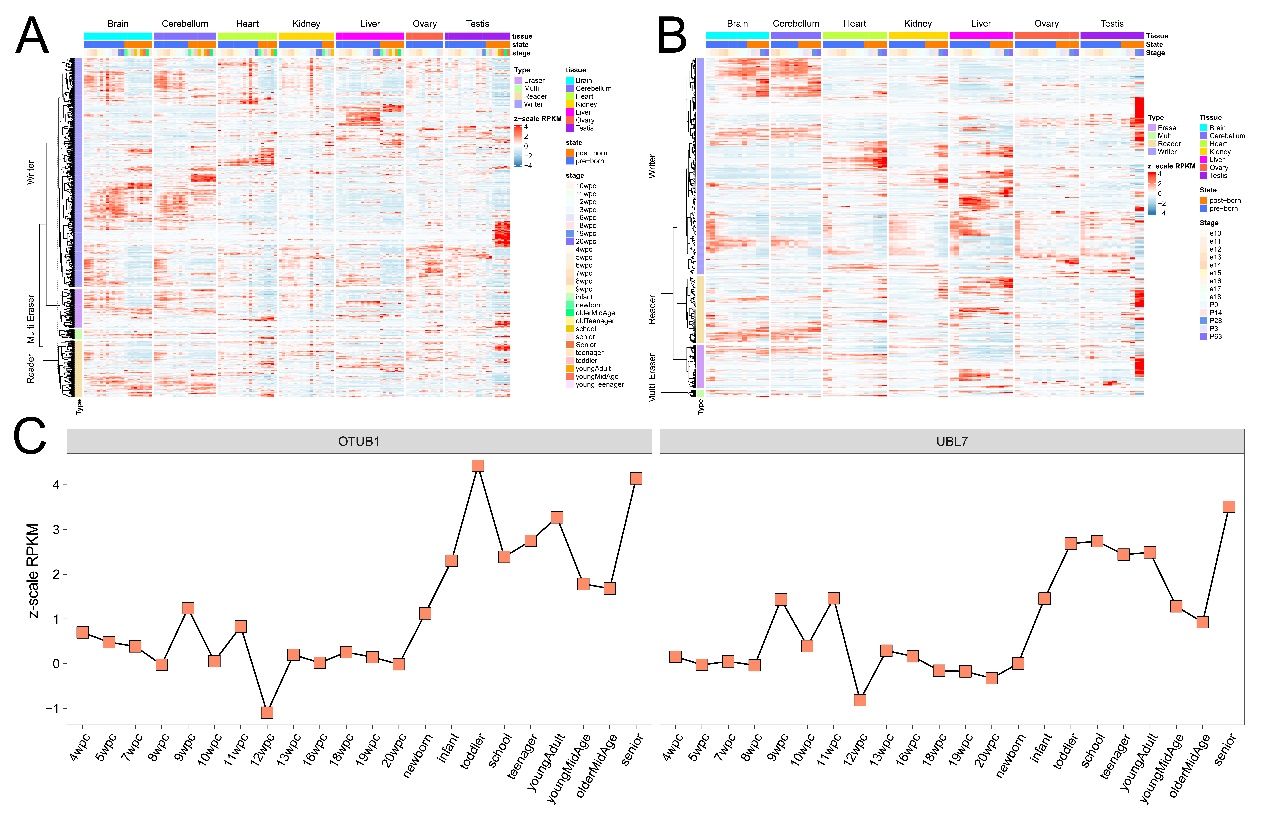


Supplementary Fig. S4. Dynamic expression of UBRs across tissue development. (**A-B**) Expression heatmap of UBRs. (**A**) human, (**B**) mouse. (**C**) Expression of *OTUB1* and *UBL7* during human brain development.


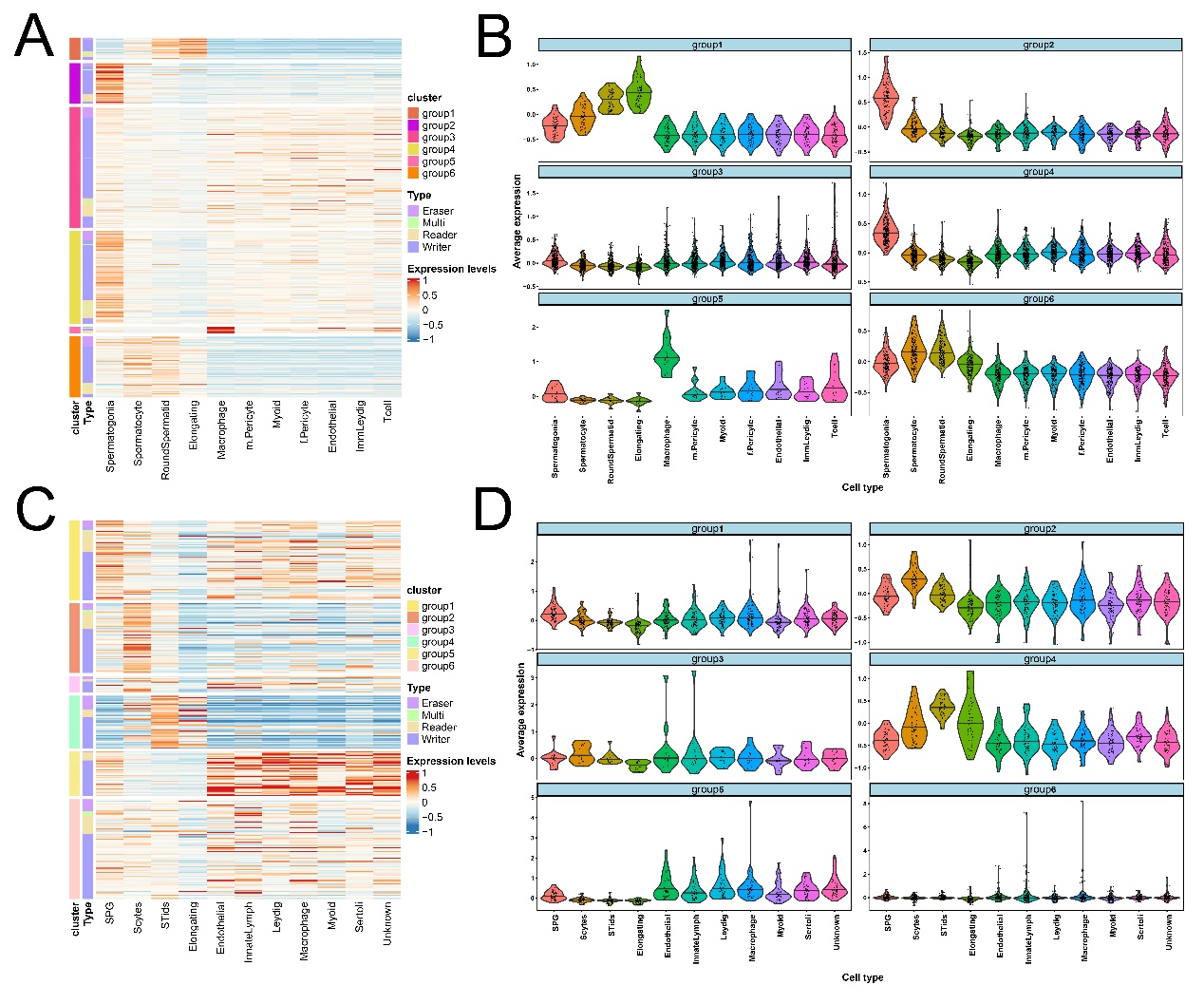


Supplementary Fig. S5. Expression patterns of UBRs in human and mouse. (**A**) Expression heatmap of UBRs in different cell types of human testis. (**B**) Changes in the expression of UBRs in different groups across cell types in human testis. (**C**) Expression heatmap of UBRs in various cell types of mouse testis. (**D**) Changes in the expression of UBRs in different groups across cell types in mouse testes.


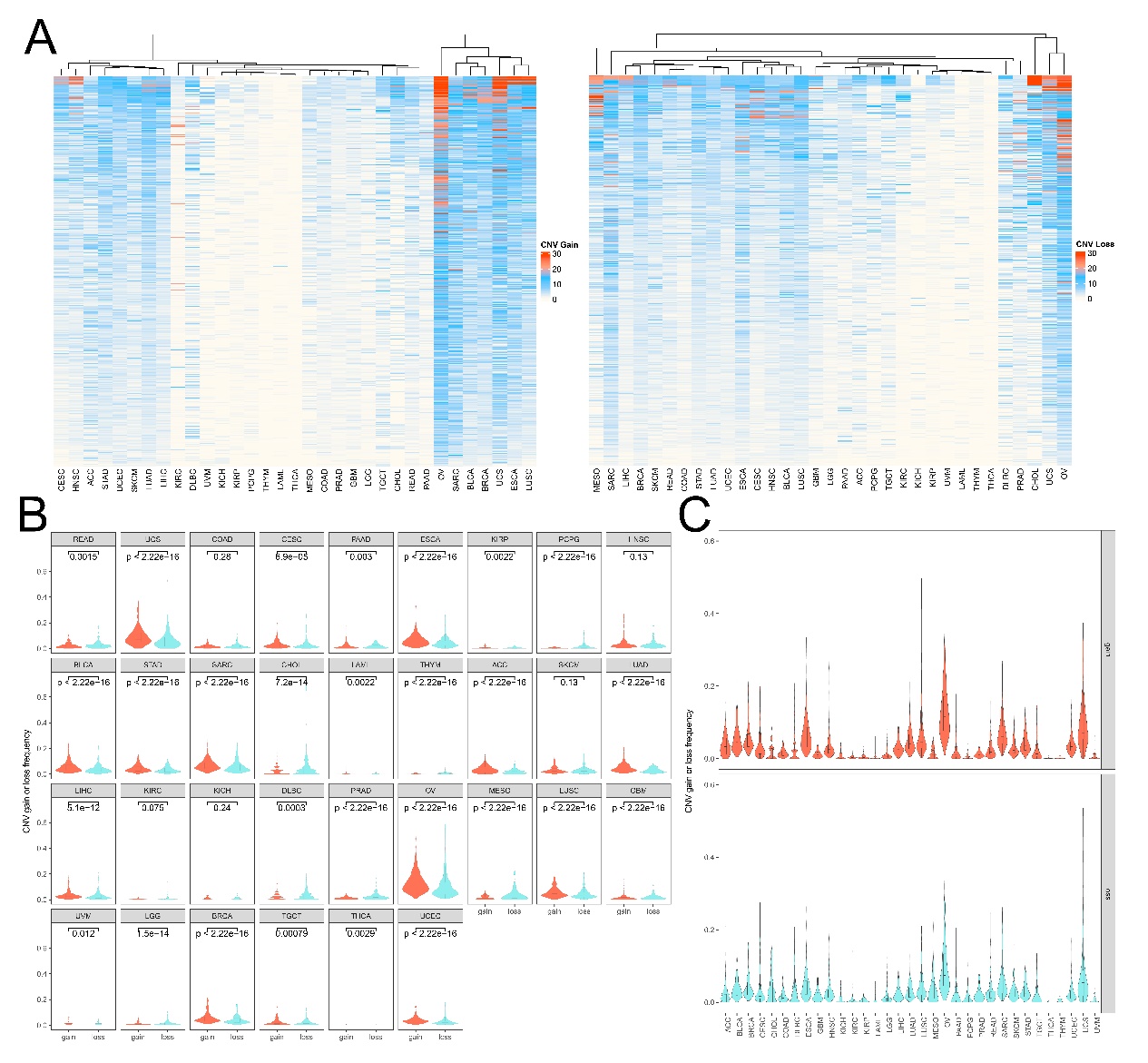


Supplementary Fig. S6. CNV gain or loss frequency of UBRs. (**A**) Heatmap shows CNV changes of UBRs. (**B**) Comparison of CNV gain and CNV loss frequency. (**C**) Violin plot showing the frequency of CNV gain and CNV loss.


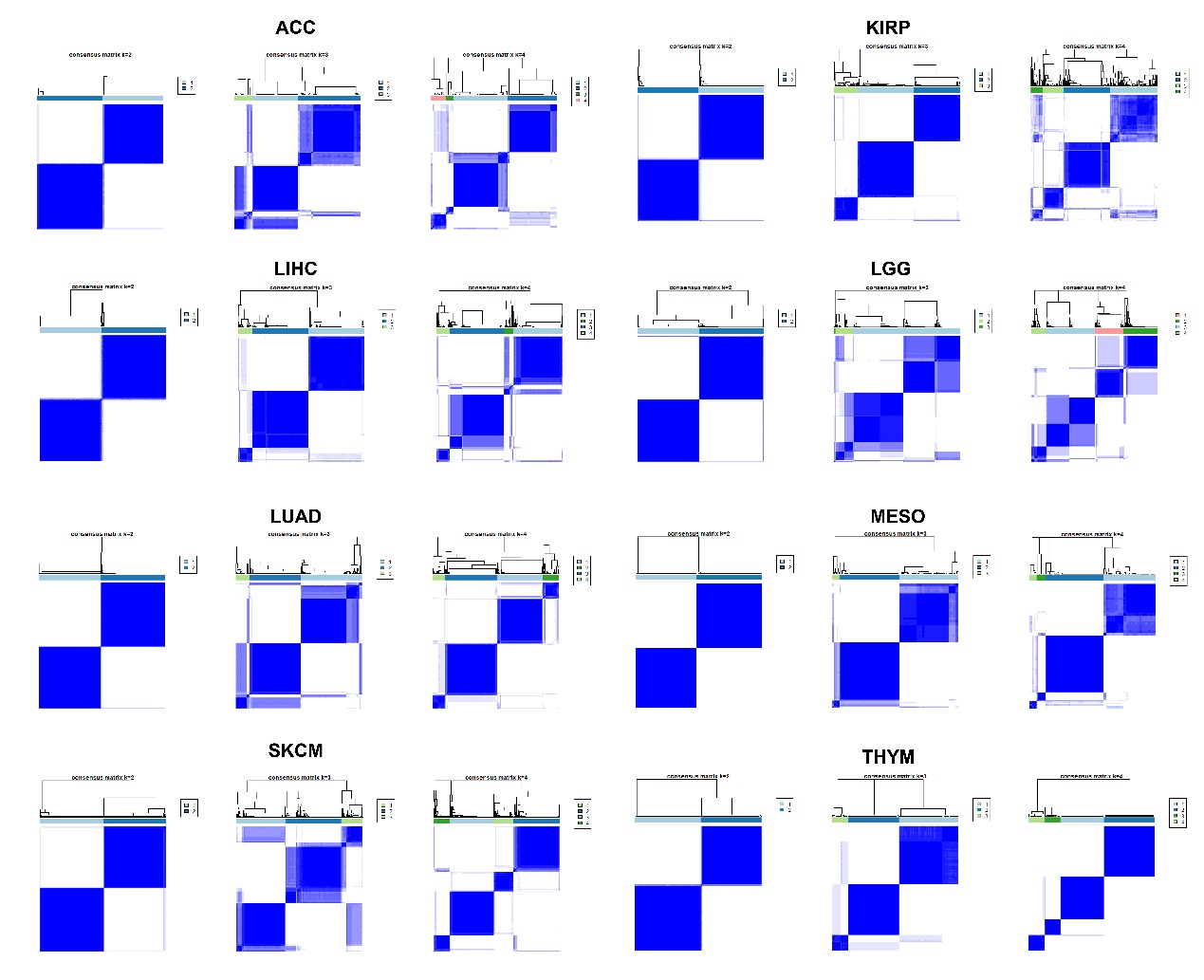


Supplementary Fig. S7. Consensus clustering analysis based on hub gene expression.


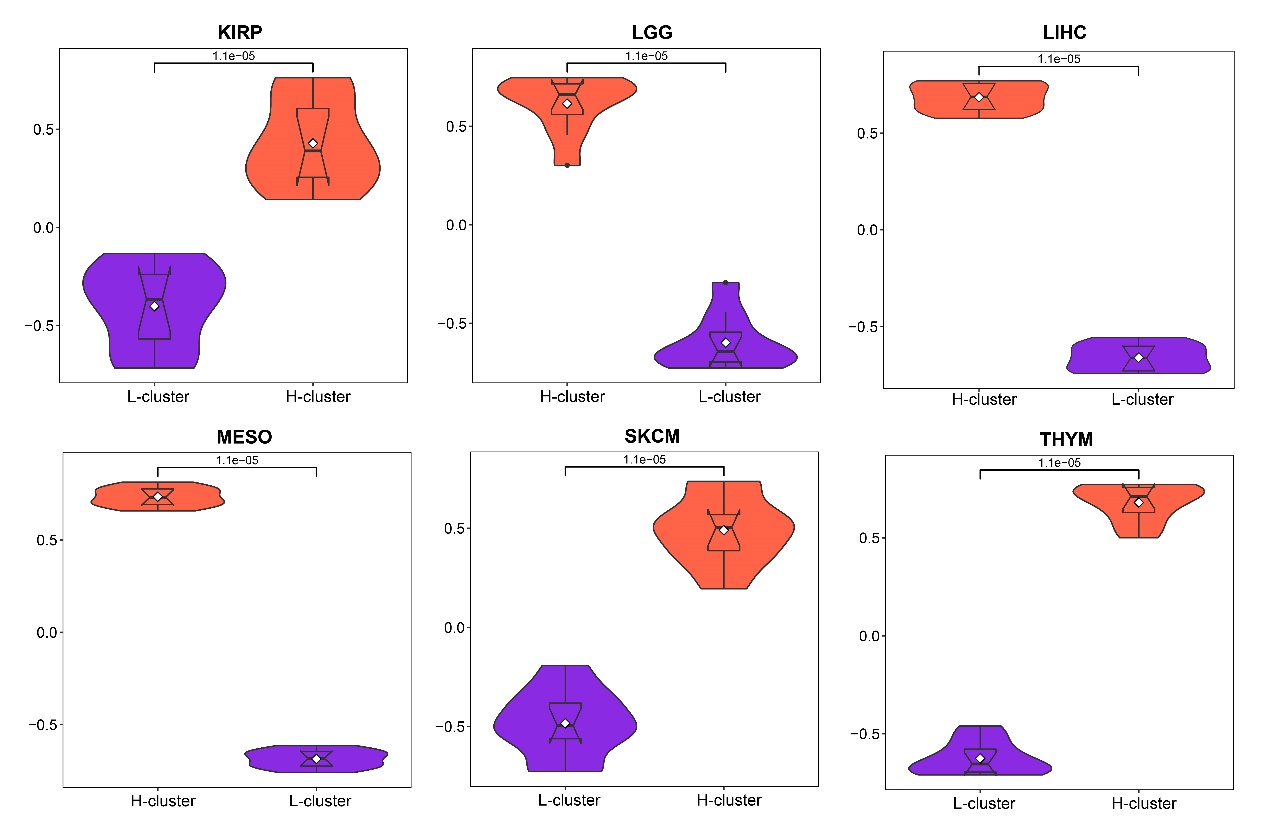


Supplementary Fig. S8. Violin plot of average expression of hub genes.


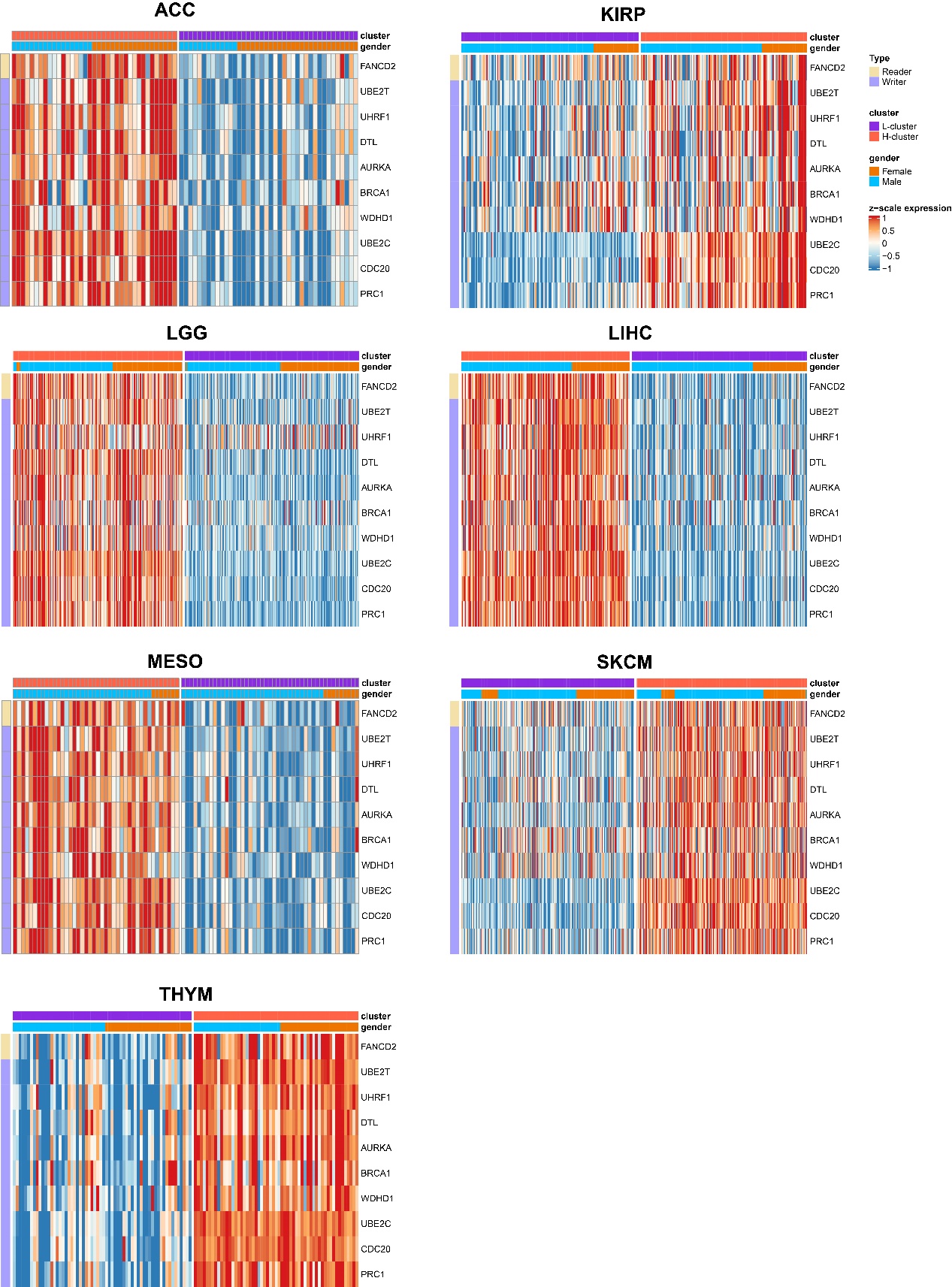


Supplementary Fig. S9. Heatmap of hub gene expression.


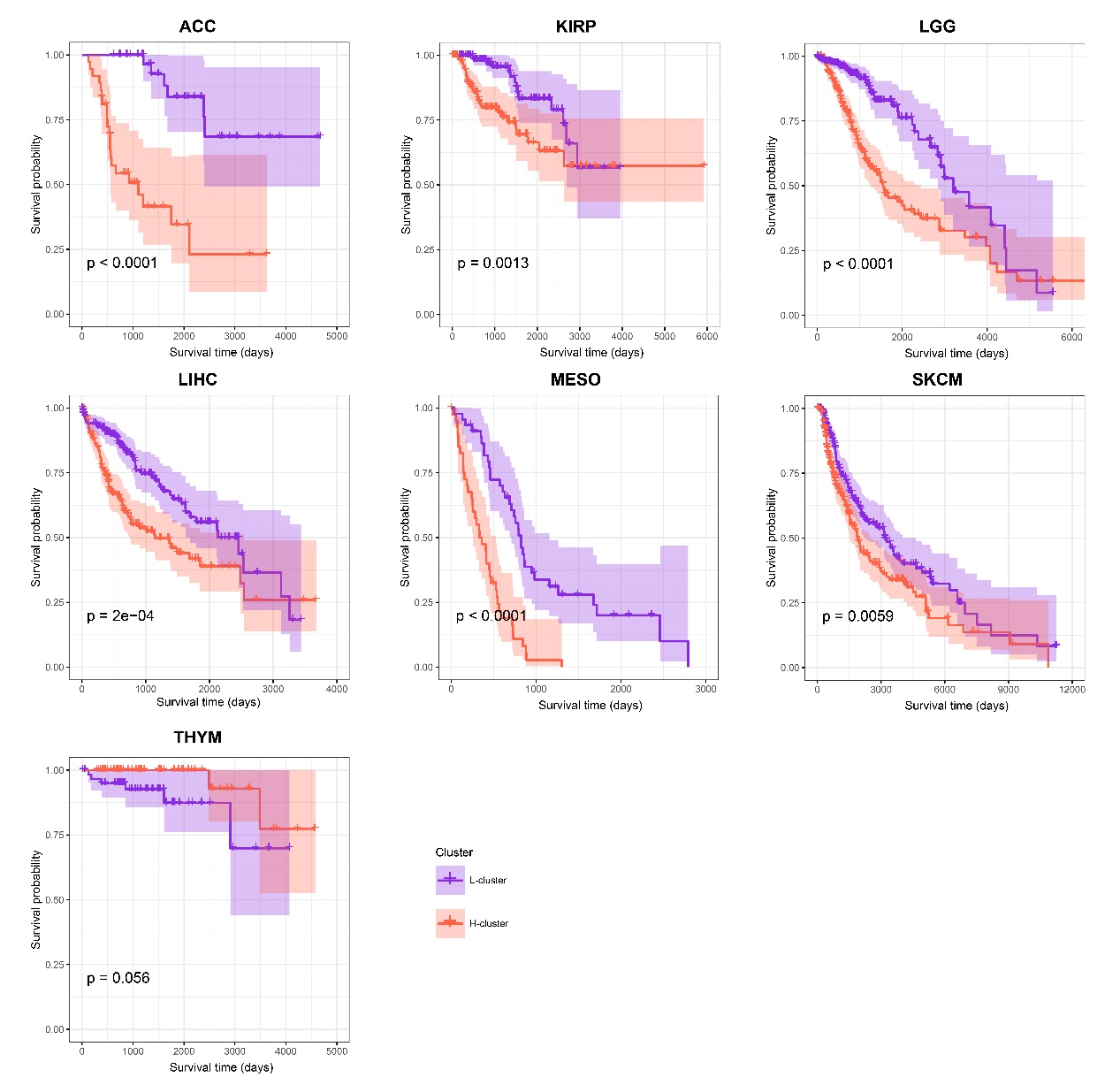


Supplementary Fig. S10. Kaplan-Meier survival plots for patients.
